# Varying genesis and landfall locations for North Atlantic tropical cyclones in a warmer climate

**DOI:** 10.1038/s41598-023-31545-4

**Published:** 2023-04-04

**Authors:** Mackenzie M. Weaver, Andra J. Garner

**Affiliations:** Department of Environmental Science, Rowan University, Glassboro, NJ 08028 USA

**Keywords:** Atmospheric science, Climate-change impacts, Projection and prediction, Natural hazards

## Abstract

Tropical cyclones (TCs) are one of the most dangerous hazards that threaten U.S. coastlines. They can be particularly damaging when they occur in densely populated areas, such as the U.S. Northeast. Here, we investigate seasonal-scale variations in TC genesis and subsequent first landfall locations of > 37,000 synthetic TCs that impact the U.S. Northeast from the pre-industrial era (prior to 1800) through a very high emissions future (RCP8.5; 2080–2100). TC genesis in the Main Development Region decreases across all parts of the season from the pre-industrial to the future, with the greatest decreases in the proportion of genesis (up to 80.49%) occurring in the early and late seasons. Conversely, TC genesis in a region near the U.S. southeast coast increases across all parts of the season from the pre-industrial to the future, with the greatest increases in the proportion of genesis (up to 286.45%) also occurring in the early and late seasons. Impacts of changing TC genesis locations are highlighted by variations in where TCs make their first landfall over the same time periods, with an increase in landfalls along the mid-Atlantic seaboard from Delaware to North Carolina during all parts of the season from the pre-industrial to the future.

## Introduction

Tropical cyclones (TCs) are some of the most destructive natural disasters that threaten coastal communities along the United States’ (U.S.) Atlantic and Gulf coasts, capable of causing billions of dollars in damages and often leading to considerable casualties. From 2017 to 2022, the U.S. was impacted by four of the costliest TCs on record in the U.S.—Hurricanes Harvey (2017), Maria (2017), Ida (2021), and Ian (2022). These four storms cumulatively cost the U.S. over $453.3 billion and caused over 3000 casualties^1–4^.

The Atlantic hurricane season officially spans from 1 June until 30 November, with the greatest number of TCs expected between the months of August and October^5^. TC genesis requires warm sea surface temperatures, low wind shear, ample humidity, adequate influence from the Coriolis force, and a pre-existing low-pressure disturbance in the atmosphere^6,7^. For these reasons, TCs have often historically formed in a region of the tropical North Atlantic known as the Main Development Region, where conditions are often ideal for TC development. In a warmer future, however, evolving TC characteristics-including their genesis, tracks, termination, and landfall patterns–may change. In particular, it is plausible that environmental changes caused by planetary warming could alter when and where TC genesis takes place, impacting where storms travel and eventually make landfall^8–13^.

Many previous TC modeling and observational studies have found that TC genesis in the North Atlantic shifts eastward under warmer climate conditions^12,14–16^, primarily due to increases in sea surface temperatures in the North Atlantic Main Development Region. However, these studies tend to focus exclusively on TCs that form in the North Atlantic Main Development Region, and/or focus on relatively short (decadal) time scales. For instance, Ref.^16^ found that under a warming climate, tropical North Atlantic TC genesis from 1950 to 2010 shifted eastward, contributing to an increase in open ocean storm tracks. Similarly, Ref.^12^ found that an eastward shift in TC genesis results in changing TC activity along the U.S. Gulf and East coasts in the 2030s. At least one long-term study has also found significant changes to the tracks of TCs that impact the U.S. Northeast, including broad spatial shifts to genesis, translation speed, and track location over time, causing storms to not only reach coastal communities more quickly, but also linger longer over key coastal locations^17^. While findings from Ref.^17^ suggest broad long-term changes to TC behavior that will be critical for the U.S. East coast, the work does not consider how seasonal-scale variation in TC behavior—including landfall locations—may evolve in a warmer climate. Here, we aim to fill this knowledge gap by performing an in-depth assessment of long-term changes to seasonal patterns of both TC genesis and landfall that may impact the crowded coastlines of the U.S. Northeast. We find changes in both spatial and temporal TC genesis trends from the pre-industrial era to the future that impact TC landfall locations and the hazard that these storms pose to U.S. Atlantic and Gulf coast communities.

## Results

We consider changes in genesis patterns in three distinct regions, including the main development region (MDR; defined here as the region within 6–18°N and 20–60°W), the Caribbean Sea (Caribbean; defined here as the region within 5–24°N and 60–90°W), and a region north of the MDR near the coast of the southeastern U.S. (SE US; defined here as the region within 25–37°N and 45–85°W). We evaluate changes in TC genesis and landfall patterns for three separate eras: the pre-industrial era (850–1800), the modern era (1970–2005), and a future era under a very high emissions scenario (Representative Concentration Pathway 8.5^18^; 2080–2100). To evaluate changes to seasonal patterns of TC genesis and subsequent landfall locations, we consider variations during three different times throughout and surrounding the Atlantic hurricane season: early season (April through July), peak season (August through October), and late season (November and December). Note that since the peak of the Atlantic TC season is temporally skewed to the later half of the full season^5^, the length of time covered during each part of the season is not evenly distributed.

When discussing our results, we use differences in percentages to discuss changing amounts of total genesis within each region relative to other regions across eras. For example, if one region had 10% of total early season genesis in the pre-industrial era, but 30% of total early season genesis in the future era, we would say the percentage of genesis in that region increased by 20% over time. We use proportions, however, to compare the change in genesis within a particular region relative to itself from one era to another. For instance, using the same region from the above example, we would say the proportion of genesis in that region increased by 200% from the pre-industrial to the future era (see “Methods”).

**Figure 1:**
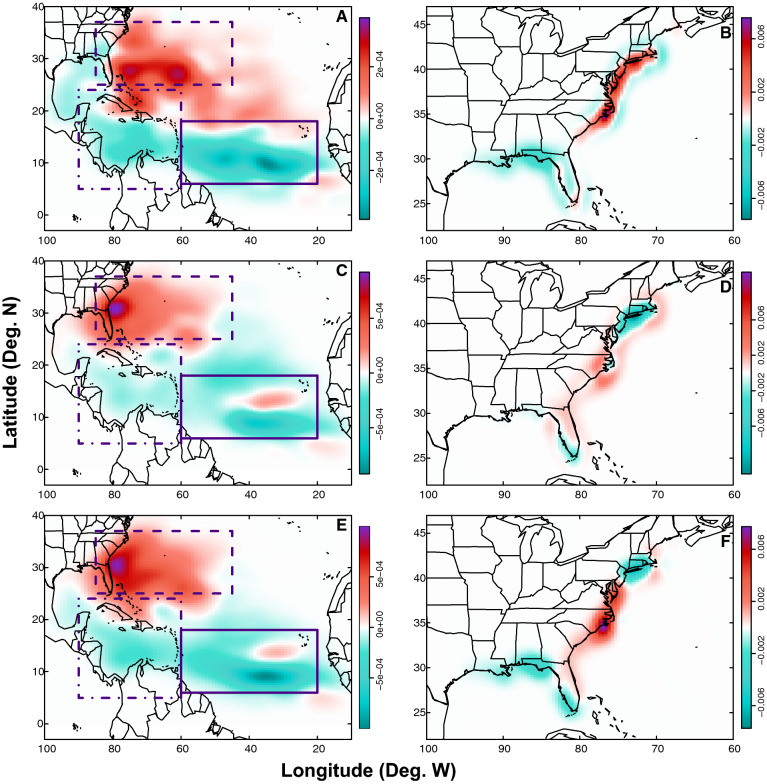
Density difference maps of TC genesis and landfall locations. Maps show the density differences of TC genesis points (**A**, **C**, **E**) and TC first landfall points (**B**, **D**, **F**). Density differences are shown for the modern era minus the pre-industrial era (**A**, **B**); future era minus modern era (**C**, **D**), and future era minus pre-industrial era (**E**, **F**). On genesis plots (**A**, **C**, **E**), solid purple rectangles designate the location of the MDR, dashed rectangles designate the location of the SE US, and dashed/dotted rectangles designate the location of the Caribbean. Note that in order to clearly show all results, colorbars are not uniform across maps. Units for the colorbars are TC points per grid cell, where each map has 100 grid cells in both the latitudinal and longitudinal directions.

Consistent with previous studies^17^, we find that from the pre-industrial to future there is a decline in TC genesis in the MDR and in the Caribbean, and a simultaneous increase in TC genesis in the SE US. This general northwestern shift in TC genesis is apparent not only when comparing pre-industrial and future simulations (Fig. 1E), but also over shorter eras, from the pre-industrial to modern and modern to future (Fig. 1A,C). Moving beyond these broad, long-term changes, we find additional seasonal-scale spatial and temporal variations in TC genesis and landfall patterns that have critical impacts for storms that threaten the U.S. Northeast coast.

### Early season genesis variations

**Figure 2:**
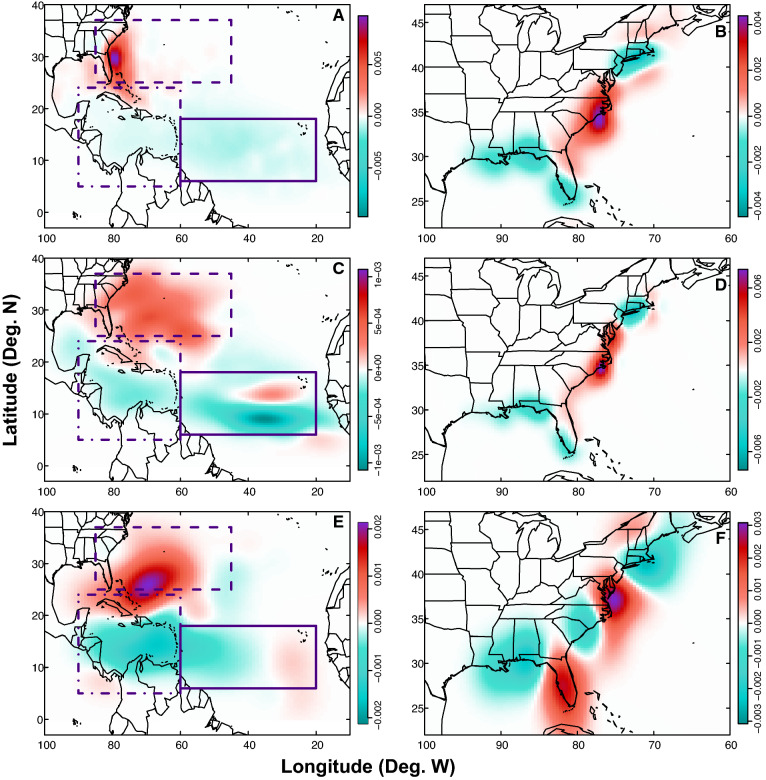
Density difference maps of TC genesis (**A**, **C**, **E**) and landfall locations (**B**, **D**, **F**) during different parts of the season. Maps show the density differences of TC genesis points (**A**, **C**, **E**) and TC first landfall points (**B**, **D**, **F**). Density differences are shown for the future minus pre-industrial early season (**A**, **B**); future minus pre-industrial peak season (**C**, **D**), and future minus pre-industrial late season (**E**, **F**). Rectangles (**A**, **C**, **E**) are as in Fig. 1. Note that in order to clearly show all results, colorbars are not uniform across maps. Units for the colorbars are TC points per grid cell, where each map has 100 grid cells in both the latitudinal and longitudinal directions.

**Figure 3:**
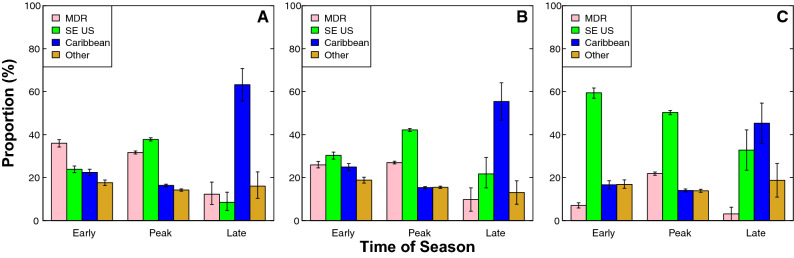
Bar graphs displaying the amount of TC genesis in each region during each part of the season. Bars show seasonal amounts of TC genesis during (**A**) the pre-industrial era, (**B**) the modern era, and (**C**) the future era. Error bars show bootstrapped 90% credible intervals.

Early season TC genesis becomes significantly more localized in the SE US from the pre-industrial era to the future (Fig. 2A). Although TC genesis is most prevalent in the MDR during the pre-industrial early season (36.03%), TC genesis is least common in this region during the early season by the future era (7.03%—a decline of ~ 29%). Genesis in the SE US (23.88%) and Caribbean (22.44%) regions are not statistically different from one another in the pre-industrial early season (90% credible interval; Table 1; Fig. 3). However, the SE US region becomes the dominant location of early-season TC genesis by the future era (59.46%—an increase of ~ 37%), far exceeding genesis in any other region during the early season (90% credible interval; Fig. 3C).

**Table 1:** Percentages of TC genesis throughout parts of the season, regions, and eras.

Part of season	Region	Pre-ind % (CI)	Modern % (CI)	Future % (CI)
Early	MDR	36.03% (34.31–37.85%)	25.90% (24.34–27.45%)	7.03% (5.77–8.29%)
	SE US	23.88% (22.34–25.41%)	30.33% (28.68–31.98%)	59.46% (57.03–61.89%)
	Caribbean	22.44% (20.91–23.97%)	24.95% (23.35–26.46%)	16.67% (14.95–18.47%)
	Other	17.65% (16.27–19.00%)	18.82% (17.36–20.28%)	16.84% (14.95–18.65%)
Peak	MDR	31.68% (30.99–32.30%)	27.00% (26.33–27.62%)	21.87% (21.06–22.71%)
	SE US	37.75% (37.05–38.45%)	42.19% (41.44–42.94%)	50.27% (49.34–51.22%)
	Caribbean	16.36% (15.80–16.87%)	15.33% (14.78–15.83%)	14.02% (13.35–14.66%)
	Other	14.21% (13.71–14.77%)	15.48% (14.92–16.02%)	13.84% (13.23–14.51%)
Late	MDR	12.26% (7.55–17.92%)	9.78% (4.35–15.22%)	3.13% (0.00–7.81%)
	SE US	8.49% (4.72–13.21%)	21.74% (15.22–29.35%)	32.81% (23.44–42.27%)
	Caribbean	63.21% (55.66–70.75%)	55.43% (46.74–63.10%)	45.31% (34.38–54.69%)
	Other	16.04% (10.38–21.70%)	13.05% (7.61–18.48%)	18.75% (10.94–26.56%)

**Figure 4:**
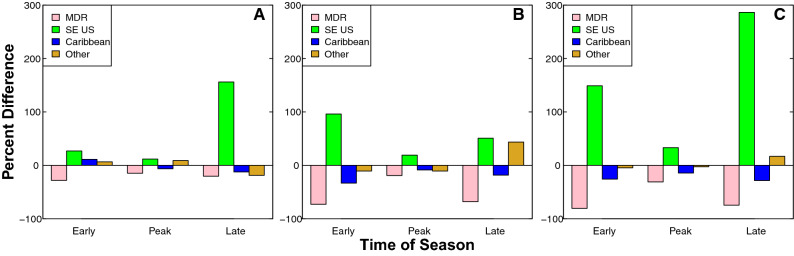
Bar graphs displaying the proportional change in TC genesis within each region during each part of the season. Bar graphs of the proportional change across (**A**) modern era compared to pre-industrial era, (**B**) future era compared to modern era, and (**C**) future era compared to pre-industrial era.

Proportional changes in TC genesis (see “Methods”) for each region further illustrate a decrease in MDR and Caribbean early-season genesis, and an increase in SE US early-season genesis from the pre-industrial era to the future. By the future era, the proportion of MDR early-season genesis decreases by 80.49% relative to the pre-industrial era. Similarly, the proportion of Caribbean early-season genesis decreases by 25.71% over the same time period. These decreases are accompanied by a 148.99% increase in the proportion of SE US genesis in the early season over the same time period (Fig. 4C).

### Peak season genesis variations

In the peak season, TC genesis variations are less extreme than in the early season, but still consistent with a general, long-term trend towards more northwestern TC genesis (Fig. 2C). There are peak-season declines from the pre-industrial to the future in both MDR (decrease from 31.68% to 21.87%) and Caribbean genesis (decrease from 16.36% to 14.02%), while there are peak-season increases in TC genesis in the SE US region (increase from 37.75% to 50.27%; Figs. 2C, 3A,C). These results are significant using a 90% credible interval (Table 1).

During the peak season, the proportion of MDR genesis decreases by 30.97% and the proportion of Caribbean genesis decreases by 14.30% from the pre-industrial to the future era. Over the same time period, the proportion of SE US genesis increases by 33.17% (Fig. 4C).

### Late season genesis variations

In the late season, genesis decreases in the Caribbean and MDR regions while increasing in the SE US (Fig. 2E). In all eras (pre-industrial, modern, and future), genesis in the Caribbean region makes up the greatest amount of late season genesis (45.31% or greater); however, by the future era, Caribbean genesis and SE US genesis are statistically similar using a 90% credible interval (Fig. 3C). Late-season genesis rates decrease in both the Caribbean from the pre-industrial (63.21%) to future (45.31%), and in the MDR from the pre-industrial (12.26%) to the future (3.13%; Fig. 3A,C). Over the same time period, TC genesis rates increase within the SE US region from 8.49% in the pre-industrial to 32.81% in the future (Fig. 3A,C). Although genesis rates in the MDR and the SE US are not statistically different from one another in the late season during the pre-industrial era, it becomes significantly more likely for TC genesis to occur in the SE US than the MDR by the late season during the future era (90% credible interval; Fig. 3C).

The proportion of late season Caribbean genesis decreases by 28.32% and the proportion of late season MDR genesis decreases by 74.47% from the pre-industrial to the future era. This decrease is accompanied by a 286.45% increase in the proportion of late season SE US genesis over the same time period (Fig. 4C).

### Changes to tropical cyclone genesis seasonal distributions

Spatial shifts in TC genesis patterns across different parts of the season coincide with changes to the temporal distributions of genesis throughout the season across the MDR, SE US, and Caribbean regions.

In the MDR and Caribbean regions, the distribution of TC genesis dates narrows from the pre-industrial era to the future, with genesis becoming more concentrated in the peak season (Figs. 6A,B,E,F, S1). For example, in the pre-industrial era, 16.41% of MDR genesis occurs in the early season, 83.31% in the peak season, and 0.28% in the late season. By the future era, the amounts of early and late season genesis decrease to 4.63% and 0.12% respectively, while the amount of peak season genesis increases to 95.25%. Though less extreme, a similar shift occurs from the pre-industrial era to the future era for the Caribbean region (Table 2). These variations result in a narrowing of the temporal distribution, with an increase in the portion of future density distribution during peak months (Fig. 6A,B,E,F).

**Table 2:** Percentages of TC genesis within each region across parts of the season and eras.

Region	Part of Season	Pre-ind %	Modern %	Future %
MDR	Early	16.41	13.81	4.63
	Peak	83.31	85.96	95.25
	Late	0.28	0.23	0.12
SE US	Early	9.85	10.71	15.12
	Peak	89.97	88.95	84.40
	Late	0.18	0.33	0.48
Caribbean	Early	18.69	20.99	14.90
	Peak	78.65	76.98	82.77
	Late	2.67	2.02	2.33
Other	Early	17.56	16.83	15.40
	Peak	81.64	82.67	83.61
	Late	0.81	0.51	0.99

Alternatively, the seasonal distribution of SE US genesis becomes broader over time, with the amount of SE US genesis becoming less concentrated during the peak season in the future compared to the pre-industrial era (Figs. 6C,D, S1). In the pre-industrial era, 9.85% of SE US genesis occurs in the early season, 89.97% in the peak season, and 0.18% in the late season. By the future era, the amounts of early and late season genesis increase to 15.12% and 0.48% respectively, while the amount of peak season genesis decreases to 84.40%. This variation results in a broader temporal distribution for TC genesis in the future, with a decreased density during peak months, and increased density during the early- and late-season months.

### Vertical wind shear and relative humidity anomalies at time of TC genesis

Shifts in TC genesis patterns are accompanied by shifts in vertical wind shear and atmospheric relative humidity. A comparison of shear and relative humidity values that provide a snapshot in time of the environment in which each TC forms allows us to examine how these variables may change for TCs forming across regions in the future relative to a 1980–2000 baseline (see “Methods”).

**Figure 5:**
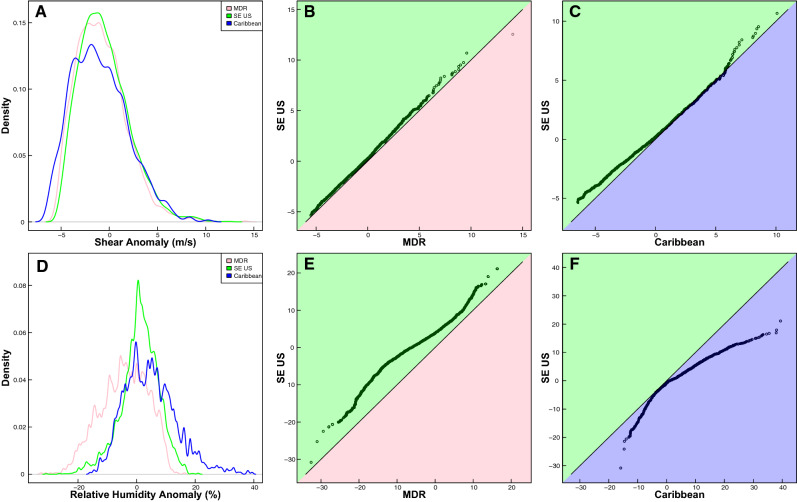
Probability density functions of vertical wind shear (**A**) and relative humidity (**D**) anomalies across regions for each genesis point in the future era relative to the 1980–2000 baseline. Note, the baseline is defined as the 1980–2000 mean of (**A**) wind shear and (**D**) relative humidity values across the full season for each individual region. In figures (**A**) and (**D**), lines represent the MDR (pink), SE US (green), and Caribbean (blue) regions. QQ-plots (**B**, **C**, **E**, **F**) compare the quantiles of the distributions of the wind shear (**B**, **C**) and relative humidity (**E**, **F**) anomalies between the MDR and SE US (**B**, **E**) and between the SE US and Caribbean (**C**, **F**). Background colors on QQ-plots are the same as colors used for PDFs, and indicate different regions. Black solid lines on the QQ-plots show the 1–1 line; points that diverge from this line indicate that the distributions for the two regions being compared are significantly different.

The distributions of 850–250 hPa vertical wind shear at the time of genesis indicate a decrease in shear across all regions by the future era, with the greatest decreases occurring in the Caribbean (Fig. S3). Throughout the season as a whole, future SE US wind shear anomalies are higher (i.e., decrease less or increase more compared to the modern baseline) relative to changes in MDR or Caribbean shear values (Fig. 5A–C). In the late season, mean shear anomalies indicate slight increases in shear values within the MDR by the future, although the mean shear anomalies across the MDR, SE US, and Caribbean regions are not statistically different from one another (Fig. S4).

Distributions of 600 hPa relative humidity values at the time of genesis indicate a notable decrease in relative humidity in the MDR in the future (Fig. S5) compared to a 1980–2000 baseline. Relative humidity distributions also indicate an increase in future relative humidity in the Caribbean and SE US regions (Fig. S5) relative to a modern baseline. Throughout the hurricane season, Caribbean relative humidity anomalies are generally similar to or higher than changes within the SE US region (i.e., Caribbean anomalies decrease less, or increase more relative to a modern baseline). However, SE US relative humidity anomalies are higher than changes in the MDR region (i.e., SE US anomalies decrease less or increase more compared to a modern baseline; Fig. S5). Throughout the early, peak, and late season, the absolute magnitude of SE US relative humidity changes are somewhat smaller than in the other two regions (Figs. 5D, S4).

### Impacts upon tropical cyclone landfall locations

Coinciding with genesis changes to TCs that impact the U.S. Northeast coast, there are variations in the locations where these TCs first make landfall along the U.S. coast.

Throughout the Atlantic TC season, the frequency of first TC landfalls increases from the pre-industrial to the modern era along the majority of the East coast from Massachusetts to South Carolina. Over the same time period, the frequency of first TC landfalls decreases in Florida and along the Gulf coast (Fig. 1B). From the modern to the future era, there are decreases in first TC landfalls along the Northeast coast from Massachusetts to New Jersey, whereas first TC landfall frequency increases along the eastern seaboard from Virginia to Florida (Fig. 1D). Comprehensively, from the pre-industrial to the future, there are long-term decreases in first TC landfalls along both the Gulf coast and the northeastern Atlantic coast between New York and Massachusetts, and long-term increases in first TC landfall frequency along the U.S. eastern seaboard from southern New Jersey to North Carolina (Fig. 1F).

At the seasonal scale, variations in landfall during the early and peak seasons indicate decreases in first TC landfalls from the pre-industrial to the future along the Gulf and Northeast coasts but increases in first TC landfalls along substantial portions of the mid-Atlantic seaboard and southeastern U.S. coastline (Fig. 2B,D). In the early season, decreases in first landfalls are observed as far south as the southern New Jersey coast (Fig. 2B).

Late season landfalls are less common owing to the smaller number of storms during this part of the season (Tables 2, 3; see “Methods”), and variations in landfall locations show a slightly different pattern. From the pre-industrial to the future, first landfall frequency still decreases along much of the Gulf coast and in the northeast during the late season; however, there are also additional decreases in first landfall frequency in North and South Carolina (Fig. 2F). Similarly, first landfall frequency again increases during the late season from the pre-industrial to the future along the mid-Atlantic seaboard from Delaware to Virginia, but additional increases are observed along Florida’s coastlines during this part of the season as well (Fig. 2F).

**Table 3:** Number of TCs in each region for each era and part of the season compared with the total number of TCs in that category.

Era	Part of season	Region	Number of TCs
Pre-industrial	Early	MDR	753
		SE US	499
		Caribbean	469
		Other	369
	Peak	MDR	3823
		SE US	4556
		Caribbean	1974
		Other	1716
	Late	MDR	13
		SE US	9
		Caribbean	67
		Other	17
Modern	Early	MDR	549
		SE US	643
		Caribbean	529
		Other	399
	Peak	MDR	3417
		SE US	5339
		Caribbean	1940
		Other	1960
	Late	MDR	9
		SE US	20
		Caribbean	51
		Other	12
Future	Early	MDR	78
		SE US	660
		Caribbean	185
		Other	187
	Peak	MDR	1603
		SE US	3685
		Caribbean	1028
		Other	1015
	Late	MDR	2
		SE US	21
		Caribbean	29
		Other	12

## Discussion

In a warming climate, TC characteristics, including where storms form and where they make landfall, will vary with changing environmental conditions. Such variations will have a substantial impact on the way these natural hazards impact U.S. coastal communities. Previous work has shown that impacts of such TC variations could include enhanced intensification along the eastern seaboard and greater flood and storm surge hazards^19–22^. Other studies indicate that TCs may also move more slowly along the U.S. East coast, amplifying hazards from TCs in the future^17,23^. The long-term study we present here adds to these previous findings by filling knowledge gaps about how TC genesis for storms that impact the crowded U.S. Northeast may vary across different parts of the hurricane season under different climates, and how those changes may impact the locations at which TCs make their first landfall throughout the season.

Overall, MDR genesis decreases across all parts of the season during all eras, with the greatest decreases occurring in the early and late seasons. Similarly, SE US genesis increases across all parts of the season and during all eras, with the greatest increases also occurring in the early and late seasons. Caribbean genesis decreases in most parts of the season and during most eras, except during the early season from the pre-industrial to the modern era, when Caribbean genesis increases. Changes in TC genesis patterns within the Caribbean are generally milder than those in the MDR and SE US regions. Regional changes in genesis patterns can broadly be described as a northwestern shift in the location of TC genesis across all parts of the season from the pre-industrial to the future.

**Figure 6:**
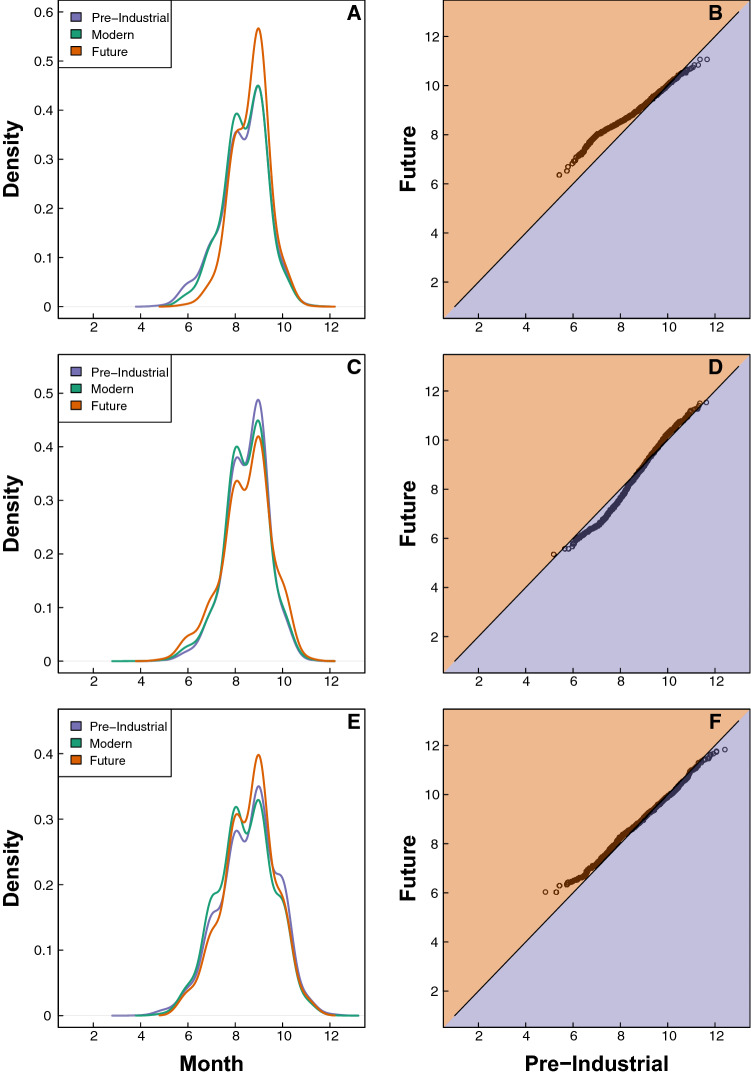
Probability density functions (**A**, **C**, **E**) and QQ-plots (**B**, **D**, **F**) of TC genesis points within each region. PDFs of (**A**) MDR genesis, (**C**) SE US genesis, and (**E**) Caribbean genesis are shown for the pre-industrial (purple), modern (green), and future (orange) eras. QQ-plots show the quantile differences of pre-industrial (purple background) and future (orange background) distributions for (**B**) the MDR, (**D**) the SE US, and (**F**) the Caribbean. Black solid lines on the QQ-plots show the 1–1 line; points that diverge from this line indicate that the pre-industrial and future distributions are significantly different.

In addition to changing probabilities of genesis, the temporal distribution of genesis throughout the season also shifts in the MDR, Caribbean, and SE US regions. While MDR and Caribbean genesis becomes more concentrated in the peak season by the future, the distribution of SE US genesis broadens. This is evident in density distributions of genesis throughout the season, which show that the density of MDR and Caribbean genesis increases in the peak season and decreases in the early and late seasons, whereas the density of SE US genesis decreases in the peak season and increases in the early and late seasons (Fig. 6). These shifts lead to an amplified seasonal cycle of TC development in the MDR and Caribbean, but a milder seasonal cycle of TC development in the SE US during future eras. In a practical sense, such variations suggest that relatively fewer TCs will develop in the MDR and Caribbean regions during the early/late parts of the season, while relatively more TCs will develop in the SE US during these parts of the season in a future warmer climate.

Environmental factors driving TC genesis variations remain somewhat uncertain. An analysis of air temperature anomalies demonstrates the well-documented rise in temperature we expect to see in a warmer future^24^, but there is no clear connection between increases of 600 mb temperatures and changes in TC genesis (Fig. S2). Sea surface temperature (SST), however, is a very important factor in TC genesis and propagation, as adequately warm SSTs provide energy to fuel TCs^6,7,25^. As both such an influential component for TC formation and a major factor in TC climatology, SST may play a role in the shifting genesis patterns demonstrated here. Previous studies provide insights into the potential impact of varying SSTs upon TC genesis behavior^26,27^. For example, the shifts in TC genesis trends within the North Atlantic basin identified here align with the projected increases in SSTs within CMIP5 models under RCP8.5 for the North Atlantic. Specifically, projected increases in September SST off the southeast coast of the U.S. from 1976 to 2099 are consistent with the increases in TC genesis we find in this region by the future era^28^. However, additional work is needed to fully examine seasonal-scale variations of SSTs over time, and the potential impact of such variations upon TC genesis patterns.

Beyond atmospheric and sea surface temperature metrics, it is plausible that changes to other environmental variables (such as wind shear or relative humidity) could also play a role in TC genesis behavior. Changes in the distributions of vertical wind shear at the time of genesis indicate a general decrease in wind shear for all three of our main regions in the future relative to the pre-industrial era (Fig. S3). This suggests that shear conditions tend to become more favorable for TC genesis in all regions in the future when considering the season as a whole. The varying magnitude of shear decreases across regions, however, cannot fully explain spatial shifts in TC genesis, given that relative changes in shear in the SE US are generally less favorable for TC genesis compared to changes within the MDR or Caribbean regions (Fig. 5B,C). Nonetheless, past studies have presented evidence that vertical wind shear limits MDR genesis in the late season^29^, consistent with the decrease in late-season MDR genesis from the pre-industrial to the future era demonstrated here. Other research suggests that in a warmer climate, vertical wind shear is also expected to increase in the lower latitudes of the North Atlantic basin and decrease in relatively higher latitudes^30,31^. Though our work explores only the instantaneous environmental wind shear at the location and time of genesis, these broader projected changes in wind shear agree with the general northwestern shift in TC genesis we describe, suggesting that further work considering broad shifts in vertical wind shear as a possible driver of TC genesis changes is warranted.

Relative humidity distributions indicate higher relative humidity values in the SE US and Caribbean during all parts of the season in the future, relative to the pre-industrial era (Fig. S5), which suggests that conditions in these regions could become more favorable for TC genesis in the future. Relative humidity distributions also indicate lower relative humidity in the MDR in the future, which suggests that conditions in this region may become less favorable for TC genesis by the future era (Fig. S5). Variations in relative humidity anomalies within the SE US and MDR are consistent with results of changing TC genesis, though connections to genesis impacts in the Caribbean region are less clear (Figs. 1, 5D–F). Though our datasets rely upon environmental relative humidity values at the specific time and location of genesis, other work focused on broader shifts in relative humidity further supports the potential impacts of this environmental variable on the genesis shifts we identify. For example, past work has presented evidence that mid-level moisture limits MDR genesis during the early season^29^, which is consistent with the decrease in early-season MDR genesis demonstrated here. Relative humidity is also expected to decrease in the lower latitudes of the North Atlantic basin in some models^31^, which agrees with results of a general northwestern shift in genesis.

Although variations in shear and relative humidity may contribute to some variations in TC genesis, neither comprehensively explains all of the spatial or temporal variations in TC genesis for storms that impact the U.S. Northeast coast. Additional research that takes a broader look at environmental variables is therefore warranted in order to better understand the role of not only vertical wind shear and relative humidity in driving changes to TC genesis climatology patterns, but also other environmental variables that could contribute to such variations, such as SSTs, or possible changes to the location of the Intertropical Convergence Zone (ITCZ)^17,31–33^, which has been indicated as a driver of elevated TC activity in the western North Atlantic when it shifts to higher latitudes^33,34^. Similarly, a weakening of the Atlantic Meridional Overturning Circulation is associated with changes in the location of the ITCZ, SSTs, vertical wind shear, and mid-level humidity that could all contribute to more favorable TC genesis conditions at relatively higher latitudes and less favorable conditions in the MDR^31^. Future analyses should particularly consider not only the profile of environmental variables throughout the atmosphere (and not only at specific altitudes), but also in-depth assessments of the broader environmental variable fields from global climate models used to downscale TC tracks.

In addition to a broader analysis of environmental variables, future work may also focus on the El Niño Southern Oscillation (ENSO) and its potential to influence genesis variations such as those we identify here. ENSO has been shown to heavily influence Atlantic TC climatology. Generally, there are fewer Atlantic TCs during El Niño years and more Atlantic TCs during La Niña years^35–38^, particularly in the MDR^30^. There is also evidence that ENSO influences TC genesis locations^39^ and the number of landfalling TCs in the U.S^40,41^. Notably, vertical wind shear and relative humidity have a substantial impact on the reduction in Atlantic TC genesis during El Niño years^42^. However, the response of ENSO to anthropogenic climate change is not well understood, so it is unclear how great a role it may play in Atlantic TC genesis patterns in a warmer future^43,44^. Future work is necessary to further our comprehension of both the effect of a warmer climate on ENSO and how these shifts may impact Atlantic TC climatology.

Despite lingering uncertainty in the environmental mechanisms driving TC genesis changes throughout the hurricane season, there are critical impacts for U.S. coastlines that arise from such changes in genesis over time. Coinciding with changes to where TCs that impact the U.S. Northeast form, there are variations in the location where these TCs first make landfall. In general, increases in TC genesis in the SE US and decreases in MDR genesis across all parts of the season (Fig. 2A,C,E) depict a northwestward shift in TC genesis. On smaller spatial scales, within individual regions (such as the MDR during the peak season, and the Caribbean during peak and late seasons), there is a general northward shift in TC genesis patterns. Variations in TC landfall patterns (Figs. 1, 2) suggest that these shifts in genesis could reduce the likelihood of TCs taking a southern trajectory through the Gulf of Mexico (e.g., Hurricane Ida 2021^45^), and increase the likelihood of TCs taking a more northward trajectory (e.g., Hurricane Irene 2011^46^) as they travel towards the U.S. Northeast, potentially resulting in more landfalls and greater impacts along the mid-Atlantic coast in a warmer climate^35^.

There are persistent increases in first landfalls along the mid-Atlantic seaboard from Delaware through North Carolina during all parts of the season and across all eras (Fig. 2B,D,F)^36–38^. This affected stretch of U.S. coastline includes a number of critically important locations spanning interests from national security to cultural history. For example, the U.S. Naval Station in Norfolk, Virginia, is the largest naval base in not only the U.S., but also the world^47^. An increase in TC activity and impacts here could therefore pose national security risks^48^. Additionally, areas with increased landfalls also include hundreds of miles of coastlines that host popular tourist destinations, important ecosystems, and cultural and historical landmarks. For instance, results suggest that the Outer Banks in North Carolina, which is home to some of the oldest resort towns in the U.S. as well as critical ecosystems^49^, will see consistent increases in TC landfalls, creating an elevated hazard for these fragile barrier islands^50^.

The hazard that TCs present to U.S. coastlines combined with the many ways that TCs may vary in a warming climate has created an ongoing and growing need to understand exactly how such variations may impact our coastal communities^24,51,52^. Our results demonstrate that seasonal scale shifts in TC genesis locations will have a substantial impact on the hazard these storms pose to U.S. coastal communities by potentially influencing the locations where these TCs make their first landfall. This creates an increasing TC hazard for portions of the U.S. East coast–a hazard that may be compounded by other factors, such as the fact that SLR in this region is rising faster than the global average^33–35^, and that TCs are becoming more damaging upon landfall^52^. These results for a high emissions future therefore illustrate not only the importance of coastal resiliency planning for U.S. coastal communities, but also the importance of mitigation measures to limit potential future TC impacts.

## Methods

### Synthetic tropical cyclones

To overcome the challenges of a short and potentially biased observational record of TCs in the North Atlantic^53,54^, this work relies upon synthetic TCs downscaled from global climate models (GCMs). Large numbers of synthetic TCs under a range of plausible past and future climates are generated using the statistical/deterministic approach described in Refs.^55,56^. These large datasets of simulated TCs are well-suited for evaluating long-term TC genesis and landfall behavior for the various climate scenarios and time periods we consider.

Datasets of simulated TCs are based upon both kinematic and thermodynamic state variables from three different Coupled Model Intercomparison Project Version 5 (CMIP5) global climate models^57^: MPI—Max Planck Institute for Meteorology, CCSM4—Community Climate System Model version 4, and IPSL—Institut Pierre Simon Laplace. The use of the MPI, CCSM4, and IPSL models allows us to perform a seasonal-scale analysis of the long-term implications of TC genesis variations originally suggested in Ref.^17^. In addition to their use in previous studies, the MPI, CCSM4, and IPSL models are also chosen because they are the only models available that contain all necessary variables to downscale TCs for the pre-industrial, modern, and future eras, thus providing historical context for modern and future changes to TC genesis in the Atlantic. We note, however, that future studies focusing on TC genesis shifts that may exist in the newer suite of CMIP6 models would also be useful.

Genesis of downscaled TCs is accomplished using a random seeding process^56^, which involves placing (or “seeding”) warm-core vortices with peak wind speeds of 12 m/s and minimal mid-level humidity anomalies in their cores throughout the Atlantic basin at all locations north of 2° latitude during all times of the year. The majority of these “seeds” fail to form TCs, due to environmental conditions that are not conducive for development, such as low potential intensity or large wind shear. However, some vortices will evolve, developing winds of at least 21 m/s (40 kts), at which point they are designated a TC^56,58–60^, and are included in our downscaled datasets. The genesis point for these TCs is simply the first point along the TC’s track (i.e., the first 2-h time-step after the seed is placed).

TC tracks are simulated using a beta-and-advection model, which relies upon the wind fields from the GCMs (850 and 250 hPa)^56^. First U.S. landfall points are determined by interpolating the 2-hourly track points to 15-min time steps, and finding the first point at which a TC crosses a U.S. state boundary.

Environmental variables for TCs are obtained from GCM fields characterizing the 600 hPa atmospheric temperature, the 850–250 hPa vertical wind shear, and the 600 hPa relative humidity of the TC’s surroundings. These variables are provided within our downscaled TC datasets^55,56^, with the only exception being that relative humidity values are not available for the pre-industrial and modern eras of the MPI model. These variables provide an instantaneous description of the environmental and atmospheric conditions surrounding the storm for each point along its track, including the genesis point.

TCs used in this work are filtered to travel within 250 km of NYC, as in Ref.^17^, ensuring that all TCs in our datasets will impact the U.S. Northeast. Our three eras of focus are defined as in Ref.^17^:

1. *The pre-industrial era (850–1800)* Climatological conditions prior to major anthropogenic influence.
2. *The modern era (1970–2005)* Recent climatological conditions.
3. *The future era (2080–2100)* Climatological conditions under additional warming due to anthropogenic greenhouse gas emissions. TCs are generated for a very high-emissions scenario (Representative Concentration Pathway 8.5; RCP8.5), which we expect to provide an upper bound on potential changes to future TC genesis and landfall locations^18^.

Datasets contain ~ 5000 TCs per model for pre-industrial and modern simulations, and over 12,000 TCs per century per model for future simulations, resulting in ~ 3000 TCs per model during 2080–2100. Such large datasets allow for meaningful statistical analyses of long-term changing TC genesis and landfall characteristics on a seasonal scale. The ratio of all simulated TCs to the total number of seeded TCs is used to determine the overall frequency in each dataset^56^.

Because TCs used here are designed to travel within 250 km of The Battery in NYC, these results are not necessarily representative of basin-wide TC genesis and landfall patterns in the Atlantic basin, but are extremely pertinent for the subset of TCs that impact the crowded U.S. Northeast coast. Additionally, many of the downscaled TCs make landfall and thus impact a wide range of other locations along the U.S. coastline, as illustrated in Figs. 1 and 2.

As with any modeling study, there are certain caveats associated with our approach. Many caveats related to the downscaled datasets or CMIP5 experiment design and individual models are provided elsewhere in the literature^55–57^. Particular caveats for this work may relate to our choice of models (reasons for which are described above), particularly since this subset of models form a relatively small ensemble that could increase uncertainty in our findings. This is especially true for relative humidity values in earlier time periods, where the variable is not available within the MPI model. Additionally, our downscaled datasets include relatively few storms in certain regions/seasons (e.g., the MDR region in the late season), providing somewhat limited sample sizes for analyses, that again increases uncertainty within our results. We nonetheless feel it prudent to include these analyses for completeness, and to gain as much insight as possible into changes in genesis and landfall throughout the hurricane season. Finally, it should also be noted that since seeded TC vortices have an initial peak wind speed of 12 m/s, any outside factors that may affect the development of these vortices prior to this point are not accounted for with the downscaling technique used here^55,56^. However, this downscaling method has nonetheless been shown to capture many aspects of observed TC variability quite well, including the global spatial distribution genesis and track density^56,59^, allowing the process to be highly useful for our goal of understanding temporal and spatial variability of TC formation and landfall on long time scales.

### Statistics and analyses

Maps illustrating spatial variations in TC genesis (Figs. 1A,C,E, 2A,C,E) and first landfall locations (Figs. 1B,D,F, 2B,D,F) were generated using a kernel density estimate for each era (and part of the season in Fig. 2). The kernel density estimates for the two eras of interest are then subtracted to find the spatial difference over time for the TC genesis/landfall points of interest. Density units are provided in terms of genesis/landfall points per grid cell, where each map has 100 grid cells in both the latitudinal and longitudinal directions. This results in a varying resolution for each map, depending on the latitude and longitude bounds, but no resolution is coarser than 1.60° longitude by 0.69° latitude.

Percentages of TC genesis are calculated using Eq. (1):

1 $$\frac{Total \, TCs \, in \, specific \, region}{Total \, TCs \, for \, that \, era \, and \, season}\times 100\%$$

Essentially, this calculation provides the amount of TC genesis that occurs in each region during a particular season and era. We first determine the total number of TCs generated during a specific part of the season, in a particular region, in each era (e.g. early season MDR genesis in the pre-industrial era), and then divide that value by the basin-wide total number of TCs in that era and part of the season (e.g. total early season genesis during the pre-industrial era regardless of region; Fig. 3).

Proportional changes in TC genesis across eras are calculated using Eq. (2):

2 $$\frac{New \, percentage -Previous \, percentage}{Previous \, percentage}\times 100\%$$

This allows us to determine the change in genesis within a particular region relative to itself across eras. We first subtract the previous percentage in a given region and part of the season from the new percentage, then divide this value by the previous percentage (Fig. 4). For example, in the early season, the MDR genesis in the pre-industrial era is 36.03%. To find the proportional change in TC genesis in the MDR during the early season, we first subtract this value (36.03%) from the percentage of early-season MDR genesis in the future (7.03%), and then divide by the original percentage (36.03%). This yields a proportional decrease of 80.49% in TC genesis in the MDR region in the future relative to the pre-industrial era.

Credible intervals (CIs) are determined by bootstrapping the original data^61,62^. For Figs. 3, S2, and S4, we generate 5000 bootstrap samples of the TC genesis locations (either MDR, SE US, Caribbean, or Other; Fig. 3), the temperature anomalies (Fig. S2), the relative humidity anomalies (Fig. S4a–c), and the shear anomalies (Fig. S4d–f) under consideration to determine the 90% CIs (p = 0.10).

The “Other” region shown in Figs. 3 and 4 and Tables 1, 2, 3 refers to storms that do not fall into one of our three primary regions, which we have included in the interest of transparency. These storms occur throughout the Atlantic basin in areas outside the focus of our study and therefore should not affect our overall results, as we focus on relative changes across our three main regions.

Probability density functions (PDFs) are used to assess the distribution of TC genesis in various regions throughout the season (Fig. 6A,C,E) and the distribution of vertical wind shear and relative humidity anomalies and values across regions (Figs. 5, S3, S5). We use PDFs to evaluate changes to these distributions within each region across eras (Figs. 6, S3, S5) and to assess overall distribution changes across regions (Fig. 5). QQ plots (or Quantile–Quantile plots) are used to analyze these same changes specifically from the pre-industrial to future era (Figs. 6, S3, S5) and to compare different regions to each other (Fig. 5B,C,E,F). These plots graph the quantiles of two distributions against each other (e.g. allowing us to compare the quantiles of pre-industrial MDR genesis throughout the season compared to the quantiles of future MDR genesis throughout the season). We include a solid one-to-one line on each figure; points that deviate from this line indicate that the two distributions being compared differ from one another.

Temperature, relative humidity, and vertical wind shear anomalies shown in Figs. 5, S2, and S4 are calculated relative to a 1980–2000 baseline, or reference time. In this case, our baseline represents the mean value of each environmental variable within each region across the whole season for the years 1980–2000. The anomalies for each era within any given region are then determined by subtracting the reference value for the region from all values of that particular variable (at the time of TC genesis). For example, to find the relative humidity anomalies in the MDR region during the future era, we would subtract the MDR relative humidity baseline (1980–2000 average) from each future value of relative humidity in the MDR, giving us a distribution of MDR anomalies for the future.

## Supplementary Information

Supplementary Figures.

## Data Availability

This work uses simulated tropical cyclones downscaled from a model developed by Kerry Emanuel (Massachusetts Institute of Technology), and described in Emanuel et al. (2006, 2008). All inputs to the tropical cyclone model come from CMIP5 datasets that are publicly available from the Earth System Grid Federation website (https://esgf-node.llnl.gov/projects/cmip5/). Downscaled fields from the tropical cyclone model are available for research purposes from K. Emanuel (emanuel@mit.edu) on request. Researchers will be asked to sign a non-redistribution agreement and to assert that the data will be used for nonprofit research only.
