# Supplementary material for: Varying genesis and landfall locations for North Atlantic tropical cyclones in a warmer climate
Source: Sci Rep. 2023 Apr 4;13:5482. doi: 10.1038/s41598-023-31545-4 (PMC10073115; doi:10.1038/s41598-023-31545-4)
Supplement: Supplementary file 1 — Supplementary Figures. [file 41598_2023_31545_MOESM1_ESM.pdf]

Varying Genesis and Landfall Locations for North Atlantic Tropical Cyclones in a Warmer Climate

Supplemental Material

Mackenzie M. Weaver[1*] and Andra J. Garner[1]

[1] *Department of Environmental Science, Rowan University, Glassboro, NJ 08028 USA*

*Corresponding Author: Mackenzie M. Weaver
Department of Environmental Science
Rowan University, Glassboro, NJ 08028 USA
E-mail: weaver53@students.rowan.edu

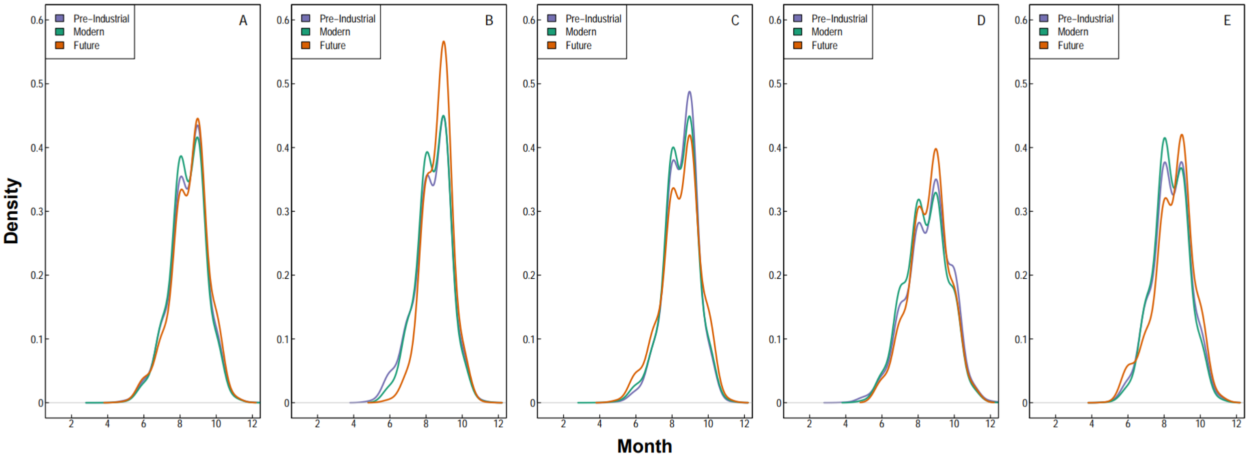

Figure S1. Probability density functions of TC genesis points throughout the Atlantic hurricane season across eras. PDFs show TC genesis for (a) all genesis, (b) MDR genesis, (c) SE US genesis, (d) Caribbean genesis, and (e) genesis outside of the three defined regions.

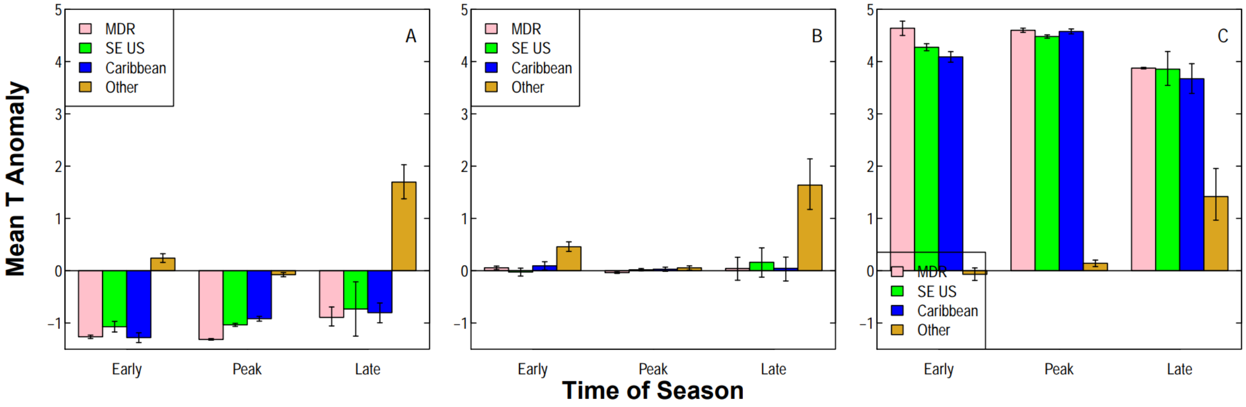

Figure S2. Bar graph of the mean temperature (T) anomalies (relative to a 1980-2000 baseline) for each region throughout parts of the season. Bars show the mean T anomaly for (a) the pre-industrial era, (b) the modern era, and (c) the future era. Error bars show bootstrapped 90% credible intervals.

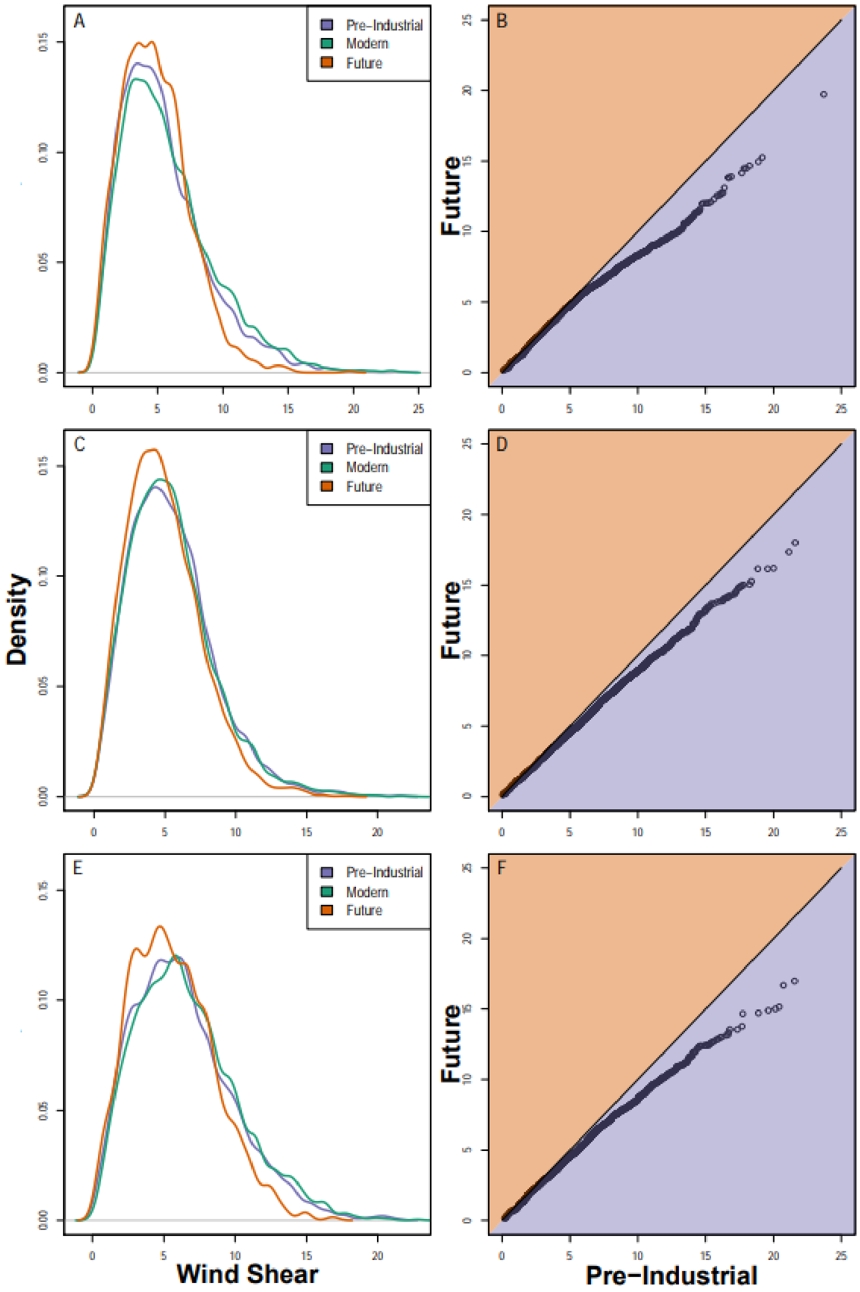

Figure S3. Probability density functions (a, c, e) and QQ-plots (b, d, f) of vertical wind shear values within each region. PDFs of (a) MDR wind shear, (c) SE US wind shear, and (e) Caribbean wind shear are shown for the pre-industrial (purple), modern (green), and future (orange) eras. QQ-plots show the quantile wind shear differences of pre-industrial (purple background) and future (orange background) distributions for (b) the MDR, (d) the SE US, and (f) the Caribbean. Black solid lines on the QQ-plots show the 1-1 line; points that diverge from this line indicate that the pre-industrial and future distributions are significantly different.

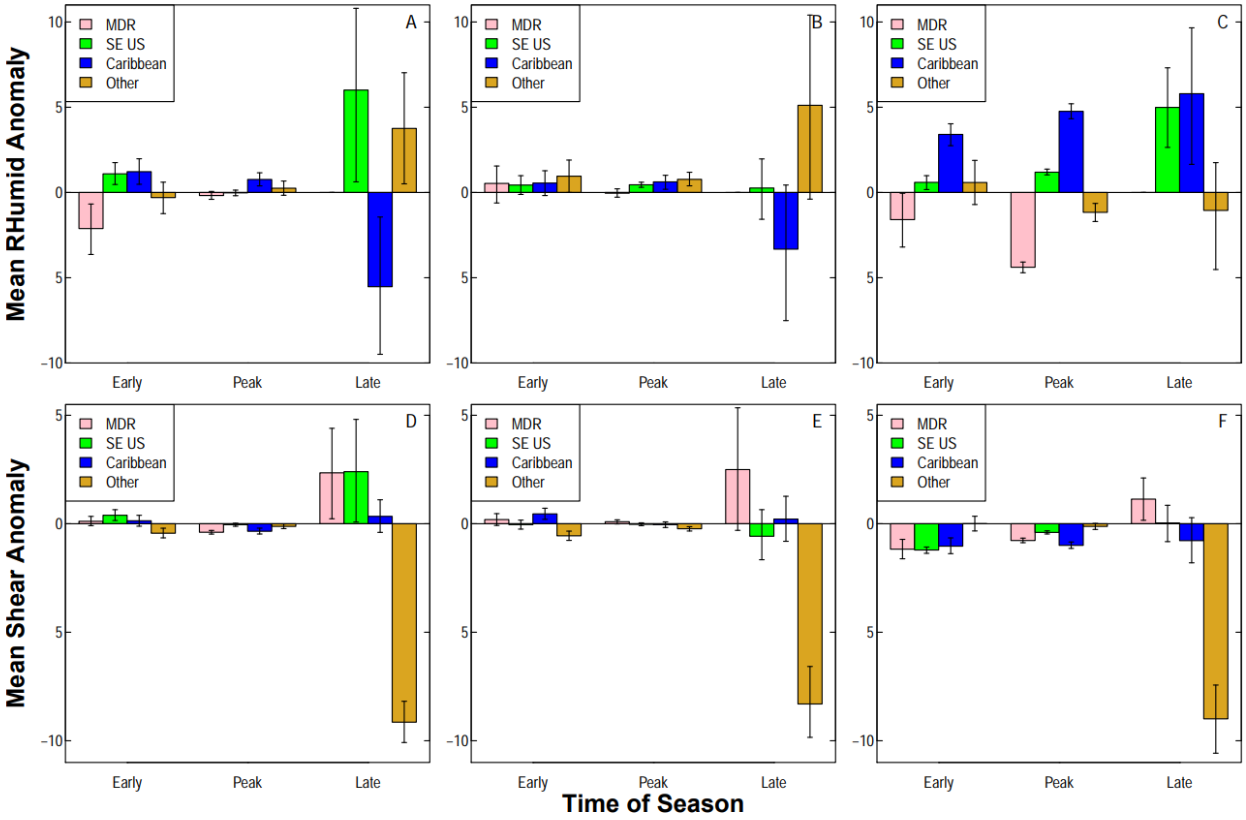

Figure S4. Bar graphs displaying the mean relative humidity (a-c) and vertical wind shear (d-f) anomalies (relative to a 1980-2000 baseline) for each region across parts of the season. Plots show the mean relative humidity anomalies during (a) the pre-industrial era, (b) the modern era, and (c) the future era, as well as the mean vertical wind shear anomalies during (d) the pre-industrial era, (e) the modern era, and (f) the future era. Error bars show bootstrapped 90% credible intervals.

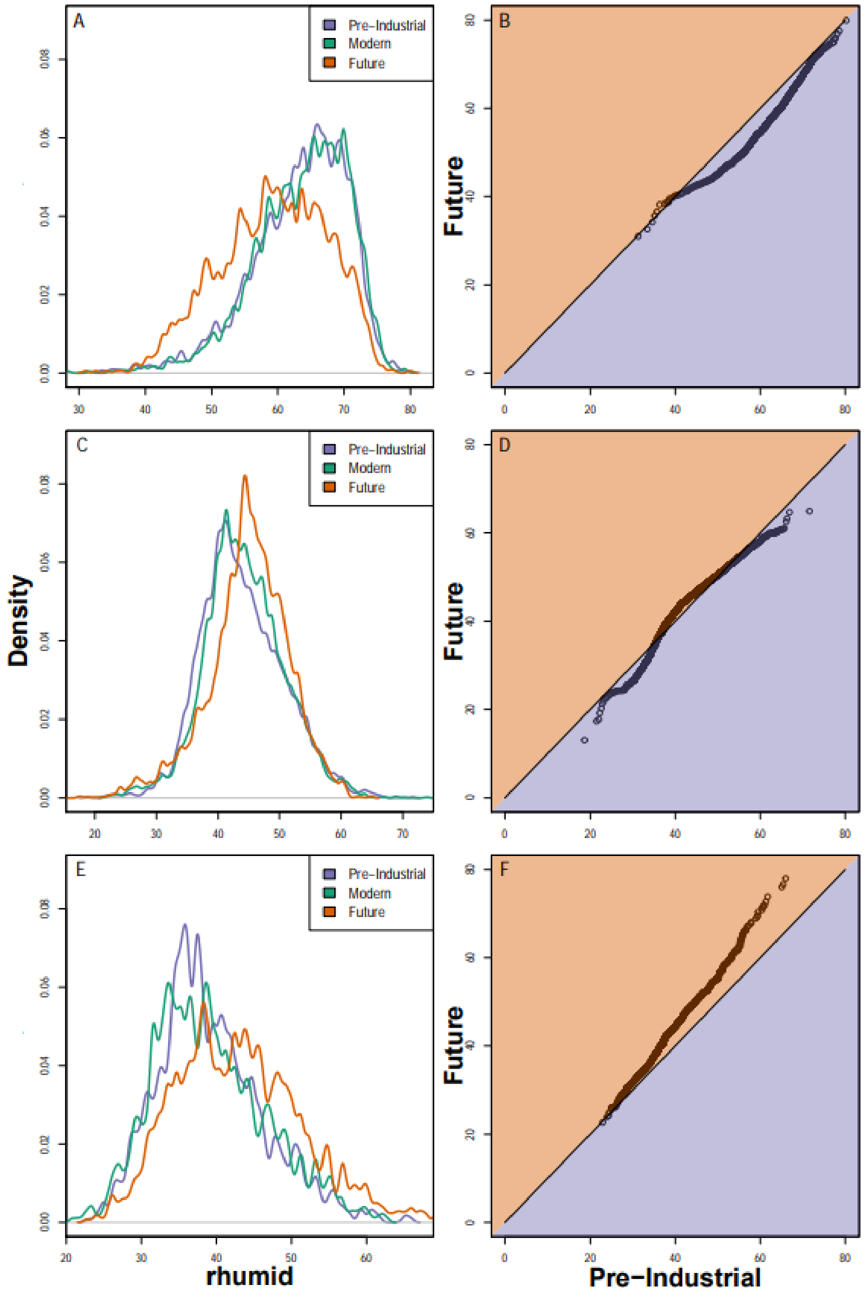

Figure S5. Probability density functions (a, c, e) and QQ-plots (b, d, f) of relative humidity values within each region. PDFs of (a) MDR relative humidity, (c) SE US relative humidity, and (e) Caribbean relative humidity are shown for the pre-industrial (purple), modern (green), and future (orange) eras. QQ-plots show the quantile vertical humidity differences of pre-industrial (purple background) and future (orange background) distributions for (b) the MDR, (d) the SE US, and (f) the Caribbean. Black solid lines on the QQ-plots show the 1-1 line; points that diverge from this line indicate that the pre-industrial and future distributions are significantly different.
